# Dual Inoculation with Mycorrhizal and Saprotrophic Fungi Applicable in Sustainable Cultivation Improves the Yield and Nutritive Value of Onion

**DOI:** 10.1100/2012/374091

**Published:** 2012-04-30

**Authors:** Jana Albrechtova, Ales Latr, Ludovit Nedorost, Robert Pokluda, Katalin Posta, Miroslav Vosatka

**Affiliations:** ^1^Department of Experimental Plant Biology, Faculty of Science, Charles University in Prague, 12844 Vinicna 5, Czech Republic; ^2^Institute of Botany of the Academy of Sciences of the Czech Republic, 25243 Pruhonice, Czech Republic; ^3^Symbiom Ltd., Sazava 170, 56301 Lanskroun, Czech Republic; ^4^Department of Vegetable Sciences and Floriculture, Mendel University in Brno, Valticka 337, 69144 Lednice, Czech Republic; ^5^Microbiology and Environmental Toxicology Group, Plant Protection Institute, Szent István University, 2100 Gödöllő, Hungary

## Abstract

The aim of this paper was to test the use of dual microbial inoculation with mycorrhizal and saprotrophic fungi in onion cultivation to enhance yield while maintaining or improving the nutritional quality of onion bulbs. Treatments were two-factorial: (1) arbuscular mycorrhizal fungi (AMF): the mix corresponding to fungal part of commercial product Symbivit (*Glomus etunicatum, G. microaggregatum, G. intraradices, G. claroideum, G. mosseae, and G. geosporum*) (M1) or the single-fungus inoculum of *G. intraradices* BEG140 (M2) and (2) bark chips preinoculated with saprotrophic fungi (mix of *Gymnopilus* sp., *Agrocybe praecox*, and *Marasmius androsaceus*) (S). The growth response of onion was the highest for the M1 mix treatment, reaching nearly 100% increase in bulb fresh weight. The effectiveness of dual inoculation was proved by more than 50% increase. We observed a strong correlation (*r* = 0.83) between the growth response of onion bulbs and AM colonization. All inoculation treatments but the single-fungus one enhanced significantly the total antioxidant capacity of bulb biomass, was the highest values being found for M1, S + M1, and S + M2. We observed some induced enhancement of the contents of mineral elements in bulb tissue (Mg and K contents for the M2 and M2, S, and S + M2 treatments, resp.).

## 1. Introduction

Soil organisms play a crucial role in the functioning of soil agricultural ecosystems. The functions performed by the soil biota have major direct and indirect effects on soil quality, crop growth and quality, its disease resistance, and thus on the sustainability of crop production systems [1]. Sustainable agriculture centres its focus on developing new comprehensive farming practices including management of soil microorganisms that are safe and environmentally friendly fostering the development of multidisciplinary studies [2].

 Among soil microorganisms, arbuscular mycorrhizal fungi (AMF) are regarded as essential components of sustainable soil-plant systems. Since the “first green revolution,” which saw the intensification of agriculture relying on high-dosage fertilization, however, less attention has been given to beneficial soil microorganisms in general and to AM fungi in particular [3, 4]. Mycorrhiza has numerous benefits for sustainable crop production. It can function as an ecological biofertilizer, a biocontrol agent against soil-borne pathogens, a bioprotectant against toxic stresses, or a soil-improver acting as a soil antierosion factor [5, 6]. Moreover, new functions of mycorrhiza in crop production are currently being explored. For example, it has been reported that AM fungi are useful for phytoremediation of contaminated soils, for example, by organophosphorus pesticides, concentration of which is high worldwide [7]. AM fungi show promising potential for reducing organophosphorus pesticide residues in plant tissues as it was shown in recent study on green onion [7].

 Native AMF populations are often reduced by soil management practices of conventional agriculture such as tillage, high-dosage use of systemic fungicides, and soluble phosphate fertilizers. These practices select against conditions favourable for the survival and development of AMF. The AMF biodiversity is often reduced in high-input systems compared to low-input ones [8, 9].

 Synergistic inoculations bring benefits to plant production, so they are already sought after. This, for example, applies to dual inoculations consisting of AMF and rhizobacteria, which promote plant-growth together [10]. Dual inoculations involving saprotrophic fungi can exhibit a beneficial effect on the growth of mycorrhizal plants. Members of the saprobic genus *Trichoderma* have emerged as an especially promising group of microbial inoculants when *Trichoderma* genotypes supporting plant growth were found [11, 12].

 Onion production in high-input cropping systems relies on high dosage of fertilizers to achieve high yields [13] since the yields are higher by almost 50% compared to organic systems, as has been documented in the Netherlands [14]. Phosphorus availability, which often determines the plant root mycorrhizal colonization and response to mycorrhiza, is usually lower in organic systems compared to conventional ones. This has been confirmed in an extensive study of onion fields in which the average phosphorus concentration (*P*_*w*_) in organic soils was up to 27% lower than in conventional soils [14]. A pioneering study on the performance of mycorrhizal onion under field conditions has shown that smaller growth benefits from mycorrhiza are observed under high phosphorus-levels than in phosphorus-deficient soils [15]. New strategies are, therefore, sought to improve phosphorus uptake and use by onion plants. Researchers focus, for example, on breeding towards improving the root system and its architecture and enhancing the responsiveness to mycorrhiza, which aids water and nutrient uptake in plants [14].

 Onion contains high amounts of a variety of antioxidants, mainly of flavonoid character (quercetin, luteolin, kaempferol, etc.), of which quercetin glycosides represent the highest portion [16]. Onion is, therefore, considered a fundamental vegetable that has been valued for its medicinal qualities since ancient times. Modern research has revealed that onion possesses antibiotic, anticarcinogenic, anti-inflammatory, and antioxidative properties [17]. Biotests suggest that a diet including onion may be beneficial for the elderly as a means of improving antioxidant status [18]. Onion extracts have been reported to be effective in treating cardiovascular diseases thanks to their hypocholesterolemic, hypolipidemic, antihypertensive, antidiabetic, antithrombotic, and antihyperhomocysteinemia effects [19].

 Recent studies document that mycorrhiza can enhance the nutritive value of crop plants, for example, increase the content of antioxidants in artichoke [20]. Leaf antioxidant content can also be increased by adding an organic fertilizer instead of a conventional nitrogen source, which simultaneously promotes soil biota activity and mycorrhizal colonization, as shown for highbush blueberries [21].

 The aim of the present study was to evaluate a possible sustainable, ecological way of producing onion using synergistic microbial treatments with mycorrhizal and saprotrophic fungi while increasing yield and maintaining or improving the nutritional quality of onion bulbs.

## 2. Materials and Methods

### 2.1. Experiment Site, Plant, and Substrate Materials

Pot experiment was located outdoors in field conditions at the Horticultural Faculty in Lednice (Location: 48°47′54.502′′N; 16°48′0.39′′E, Czech Republic) in 2009. Seeds of *Allium cepa *L. (Alliaceae) cv. “ALICE” (SEMO a.s.) were sown on 24th February, and seedlings were planted into experimental 10 litre pots on 23rd April. For both the sowing and the pot experiment, we used a sterile substrate mixture composed of zeolite : peat : bark chips: bentonite (3 : 3 : 2 : 1; v/v/v/v). Substrate sterilization was done by gamma radiation (min· 25 kGy, company Artim s.r.o., Prague, CZ, http://www.artim.cz/). Basic chemical and physical properties of the substrate were 75.76% of dry matter, 14 647 mg·kg^−1^ of K, 533 mg·kg^−1^ of P, 1 159 mg·kg^−1^ of N, 0.015 mg·kg^−1^ of S, 1 250 mg·kg^−1^ of Mg, 2 536 mg·kg^−1^ of Ca, and pH 4.47. Before seedlings were planted, the substrate was fertilized, based on a chemical analysis, with 660 mg of P_2_O_5_ and amended with 5.4 g of CaCO_3_ per pot to achieve pH = 6.4. The electric conductance of the substrate was 0.18 mS·m^−1^.

### 2.2. Experimental Design with Microbial Treatments

Microbial treatments consisted of combination of two factors: (1) an AM fungal treatment (AMF)—either a mixed *Glomus *sp. inoculum (M1) or a single-isolate (M2); (2) bark chips preinoculated with saprotrophic fungi (S). The treatment M1 was a mixture of AM fungi corresponding to the composition of a fungal part of a commercial product Symbivit (Symbiom s.r.o., http://www.symbiom.cz/): *Glomus intraradices *BEG140, *G. mosseae *BEG95, *G. etunicatum *BEG92, *G. claroideum* BEG96, *G. microaggregatum *BEG56, and *G. geosporum* BEG199, only without the bioadditives that are used in the standard commercial product. The treatment M2 was a single-fungus inoculum of *G. intraradices* BEG140. The treatment S represented bark chips preinoculated with saprotrophic fungi. Saprotrophic fungi effective in wood decomposition (*Gymnopilus* sp. isolate IZO24, *Agrocybe praecox* isolate AER1, and *Marasmius androsaceus* isolate MAN1) were inoculated on nonsterile pine bark chips (chip size 7–15 mm, TerraSan, http://www.terrasan.de/online/index.php) in a mixture (1 : 3.3, v/v) and were left to grow for 3 months at 25°C. The AER-1 strain was derived from a fruit body in 2005 and identified based on morphological characteristics and ITS rDNA sequence similarity to AM905094 (derived from an *A. praecox* sporocarp) and AY194531 (derived from an *A. praecox* sporocarp, voucher MSC 378486). The final treatments tested were Ctrl (control, nontreated plants), M1, M2, S and S + M1, S + M2 combinations of two mycorrhizal inoculants with saprotrophic amendment. Each of 6 treatments involved 7 pot replicates, each pot contained 3 seedlings.

Fungal inocula were supplied by the company Symbiom s.r.o. The mycorrhizal inoculum (M1 or M2) was added as a mixture of the cultivation substrate, colonized roots, and mycelium fragments (grown as single AMF cultures on maize as the host plant for 5 months in zeolite) in a dose of 120 g into each planting hole approx. 3 cm below each seedling. Bark chips preinoculated with saprotrophic-fungi (S) were added to the bottom of cultivation pots in the amount of 500 mL mixed with 2.5 L of cultivation substrate per 10 L pot (in S treatments thus replacing part of the cultivation substrate by uninoculated bark chips). Plants were regularly fertilized with the leaf fertilizer Wuxal super (containing 98 g/L N, 98 g/L P_2_O_5_, 73 g/L K_2_O and trace elements B, Fe, Cu, Mn, Mo, Zn in physiological concentrations; product of AgroBio s.r.o. Opava, CZ, http://www.agrobio.cz/intro/) starting 8 weeks after planting (the fertilization dates: 08/06, 20/06, 02/07, 13/07, 22/07, 19/08).

### 2.3. Harvests, Plant Analyses, Mycorrhizal Parameters

The plants were harvested, measured, and sampled for plant analyses after 4 months of cultivation at 21st August 2009. The dry mass values of bulbs, shoots, and roots were recorded after drying to constant weight at 105°C in a drying oven Sterimat 574.2 (BMT, Czech Republic) for at least 24 h. Roots of all plants were sampled to determine AM colonization. All onion bulbs were sampled for analysis of antioxidant capacity and contents of mineral elements (a 10 g sample for each), and mixed 10 g-samples per treatment were prepared for the vitamin C content analysis.

### 2.4. Antioxidant Capacity Determination FRAP Assay

Onion bulb samples (10 g) were homogenized in a blender with 30 mL ethyl alcohol (50% concentration), homogenate was added with ethyl alcohol (50% concentration) to amount of 50 mL, filtered, centrifuged (3800 rt/min for 10 minutes), and the supernatant was used for the measurement. Total antioxidant capacity (TAC) was determined using the ferric-reducing antioxidant power (FRAP) assay developed by Benzie and Strain [22]. In the FRAP assay, reductants (“antioxidants”) present in the extract reduce Fe(III)-tripyridyltriazine (TPTZ) complex to its intensely blue ferrous form with an absorption maximum at 593 nm. The working FRAP reagent was prepared fresh on the day of the analysis by mixing acetate buffer, 10 mM TPTZ solution, and 20 mM ferric chloride solutions in the ratio of 10 : 1 : 1 (v/v/v). The mixture was incubated at 37°C. The absorbance was monitored for 4 min in a temperature-controlled cuvette held at 37°C using a JENWAY 6100 spectrophotometer (AIR, UK).

 The final total antioxidant capacity was expressed in mg equivalent of Trolox in 100 g of fresh biomass (mg Trolox·100 g^−1^). For quantification, a calibration curve of Trolox was prepared with dilutions from 50 mg/L to 700 mg/L.

### 2.5. Contents of Mineral Elements

Contents of Na, K, Ca, and Mg were determined by the method of capillary isotachophoresis using the IONOSEP 2003 device (RECMAN, CZ) following method described by Blatny et al. [23]. Onion bulb samples (10 g) were homogenized and diluted with distilled water (1 : 40) and then analysed. The head of electrolyte in the analysis was 5 mL mM H_2_SO_4_ + 7 mM-18-crown-6 + 0.1% HPMC^1^(hydroxypropyl methylcellulose). The terminating electrolyte was 10 mM BTP^1^ (bis-tris propane). The drive current was 100 *μ*A at the beginning and 50 *μ*A at the end. The amount of each mineral element was expressed as mg per kg of onion bulb fresh weight.

### 2.6. Vitamin C—Ascorbic Acid (AA) Content

The concentration of vitamin C (ascorbic acid) was determined only in compound 10 g samples combined from bulbs harvested in each treatment by HPLC according to Arya et al. [24] with slight modification. Onion bulb samples were homogenized in a blender with 75 mL of 0.1 M oxalic acid. The homogenate was topped up with oxalic acid to the volume of 100 mL, filtered, and centrifuged (3800 rt/min for 10 minutes), and the supernatant was used for measurement. The analyses were performed by RP-HPLC in a LCO-101 column placed in an Ecom thermostat (*t* = 30°C), mobile phase TBAH (tetrabutylamonium hydroxide) : 0.1 M oxalic acid : water in the ratio of 10 : 20 : 70 (v/v/v), flow 0.5 mL/min at 254 nm using a UV-VIS detector. The amount of AA was expressed as mg·100 g^−1^ of fresh weight.

### 2.7. Mycorrhizal Colonization

Mycorrhizal root colonization was evaluated in root samples taken from root systems of experimental plants (3 samples corresponding to 3 plants per pot). Samples were stained with 0.05% trypan blue in lactoglycerol [25] and quantified by the modified grid-line intersect method [26] using an ocular grid at a 100x magnification.

### 2.8. Statistical Analysis

We analysed the results of our experiments using Statistica 9.0 software (StafSoft Inc. 1984–2009). We tested the data for normal distribution and homogeneity of variance by Bartlett's test. The effects of experimental factors were evaluated by the analysis of variance (ANOVA), and comparisons between means were carried out using Tukey HSD test at the significance level of *P* < 0.05. Data on root colonization were arcsine/logarithmically transformed in order to meet the requirements of ANOVA prior to the statistical analysis. Measure of variability of a mean value throughout the text is expressed by ±SD. Linear correlation was evaluated with Pearson coefficient *r*, *P* < 0.05.

## 3. Results

**Figure 1:**
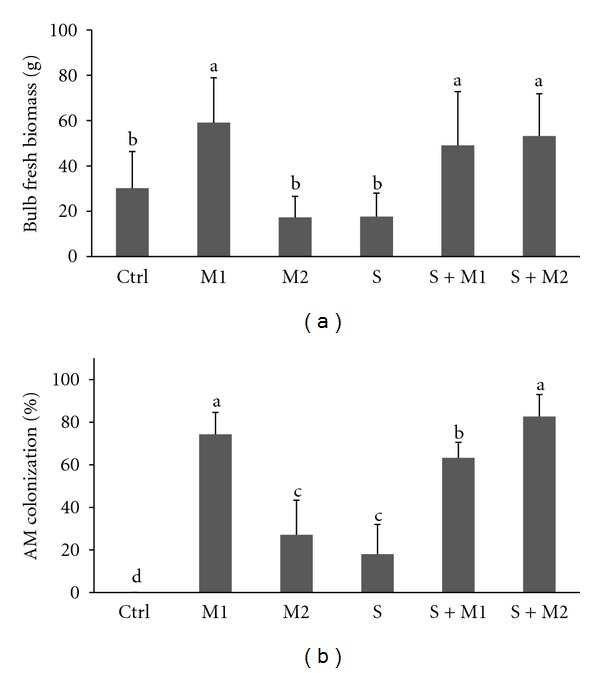
Response of onion to inoculations: (a) bulb fresh biomass, (b) AM colonization. Treatments: Ctrl—Control, M1—the mix of *Glomus* sp. (*G. intraradices* BEG140, *G. mosseae* BEG95, *G. etunicatum* BEG92, *G. claroideum* BEG96, *G. microaggregatum *BEG56, *G. geosporum *BEG199), M2—*G. intraradices* BEG140, S—saprophytic fungi preinoculated bark chips (*Gymnopilus* sp. IZO24, *Agrocybe praecox* AER1, *Marasmius androsaceus* MAN1), S + M1, S + M2. Means ± SD, columns marked with the same letters are not significantly different at the level *P* < 0.05, Tukey HSD Test, *n* = 19.

Onion growth measured as the bulb fresh biomass was significantly enhanced by three experimental inoculation treatments (Figure 1(a)).

Bulb fresh weight was the highest for the M1 mix treatment, reaching nearly a 100% increase in bulb fresh biomass comparing to control. The effectiveness of dual inoculation in both treatments (S + M1, S + M2) was proved by a more than 50% significant increase comparing to control. The lowest bulb yield was in the control and in the treatment inoculated with the saprotrophic fungus or the *G. intraradices* M2 inoculum.

**Figure 2:**
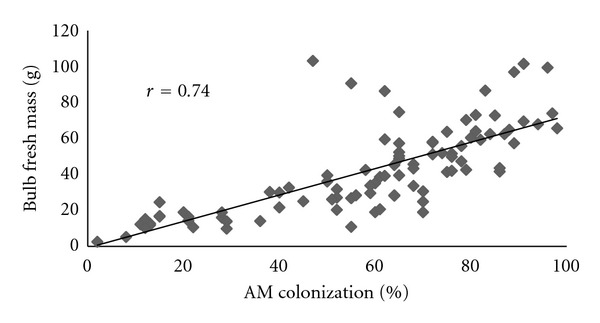
Relationship between root AM colonization (%) and bulb fresh weight (g) for all plants in inoculated treatments (M1, M2, S, S + M1, S + M2) expressed as linear correlation, *r*—Pearson correlation coefficient, *P* < 0.05, *n* = 92.

 Roots of onion plants of all inoculated treatments were colonized with the exception of the control, noninoculated onion plants (Ctrl). As expected, the highest rate of colonization was found for the treatments that involved mycorrhizal inoculation (M1 74.3 ± 10%, M2 27.2 ± 16.2%, S + M1 63.3 ± 7.3% S + M2 82.6 ± 10.4%) (Figure 1(b)). The observed AM colonization of plants inoculated with saprobes only (S) was rather unexpected, but AM colonization caused by airborne mycorrhizal propagules could not been fully prevented since the experiment was kept outdoor. Mycorrhizal colonization enhanced the yield response, and a strong correlation (*r* = 0.83, *P* < 0.05) was observed between AM colonization and the onion bulb growth (Figure 2).

**Figure 3:**
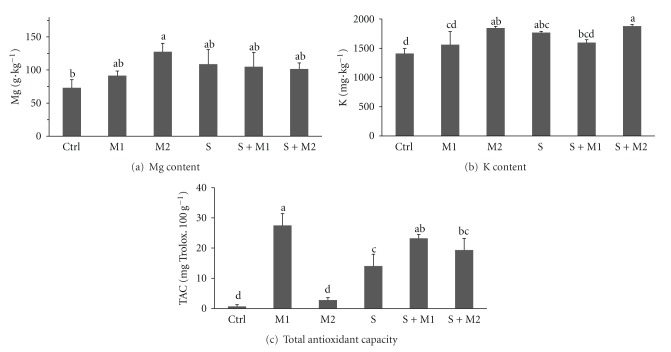
Contents of mineral elements Mg (a), K (b) and total antioxidant capacity determined by FRAP method (c) in onion bulb mass. Treatments: Ctrl—Control, M1—the mix of *Glomus* sp. (*G. intraradices* BEG140, *G. mosseae* BEG95, *G. etunicatum* BEG92, *G. claroideum* BEG96, *G. microaggregatum* BEG56, and* G. geosporum *BEG199), M2—*G. intraradices* BEG140, S—saprophytic fungi preinoculated bark chips (*Gymnopilus* sp. IZO24, *Agrocybe praecox* AER1, *Marasmius androsaceus* MAN1), S + M1, S + M2. Means ± SD, columns marked with the same letters are not significantly different at the level *P* < 0.05, Tukey HSD Test, *n* = 7.

Regarding the nutritional value of plants under different inoculation treatments, the values were either positively affected by the inoculation treatment or were not changed significantly. All inoculation treatments, but the single-fungus, (M1, S, S + M1, S + M2) enhanced significantly the total antioxidant capacity of bulb mass (Figure 3(b)). The highest values were found for the mix M1 (27.5 ± 3.98 mg Trolox.100 g^−1^) and both dual inoculations (S + M1 and S + M2 were 23.2 ± 2.51 and 19.3 ± 3.48 mg Trolox·100 g^−1^, resp.). The *G. intraradices* inoculum itself did not enhance TAC but did cause a TAC increase when combined with saprotrophic fungi. The contents of nutritionally important elements in bulb tissue were enhanced in response to the microbial treatments with only some significant values. The Mg content (Figure 3(a)) was significantly increased in bulbs under the M2 treatment (127.6 ± 12.8 mg·kg^−1^) compared to the control plants (Ctrl 73.1 ± 12.4 mg·kg^−1^). In the case of K content (Figure 3(b)), significantly increased values were found in treatment combinations involving of the AMF mix inoculum and in saprotrophic treatments (M2 1411 ± 84 mg·kg^−1^, S 1769 ± 22 mg·kg^−1^, S + M2 1879 ± 25 mg·kg^−1^), compared to the control plants (Ctrl 1411 ± 84 mg·kg^−1^). The contents of Ca and Na were not found to be affected significantly by the inoculation treatment and reached on average 46.5 ± 13.5 and 18.7 ± 5.9 mg·kg^−1^, respectively. Values of Ca content for individual treatments were Ctrl 35 ± 19.0 mg·kg^−1^, M1 61 ± 7.7 mg·kg^−1^, M2 35 ± 14.5 mg·kg^−1^, S 55 ± 4.8 mg·kg^−1^, S + M1 48 ± 5.7 mg·kg^−1^, S + M2 44 ± 5.1 mg·kg^−1^, and the values of Na content for individual treatments were Ctrl 19 ± 2.5 mg·kg^−1^, M1 20 ± 3.4 mg·kg^−1^, M2 16 ± 8.1 mg·kg^−1^, S 22 ± 4.8 mg·kg^−1^, S + M1 20 ± 3.8 mg·kg^−1^, and S + M2 15 ± 10.6 mg·kg^−1^. The content of vitamin C in onion was not found to be affected by the inoculation treatments, and its values ranged from 11 to 20 mg·kg^−1^ with the average value of 15.1 ± 4 mg·g^−1^.

## 4. Discussion

*Allium* species and *A. cepa *in particular are regarded as highly AMF responsive plants [27]. Some of the reported increases are much higher than that observed in our study, which was only twice as high for the treatment with the M1 mix inoculum compared to control, uninoculated plants. For *A. cepa,* Hayman and Mosse [28] recorded an up to 18-fold increase in the weight of mycorrhizal plants compared to nonmycorrhizal ones. Onion has a coarse root system without root hairs [29]. Such plants are often obligate mycorrhizal crops that are unable to complete their life cycle in the absence of AMF because of insufficient P uptake and hence insufficient growth [30, 31]. Mycorrhiza helps plants with such a shallow sparse root system to increase phosphorus uptake. AM fungi are, to different degree, capable of promoting phosphorus availability by acidifying the soil and, consequently, exploiting the phosphorus in nutrient patches and by facilitating the growth and development of host plants [32]. The observed high mycorrhizal responsiveness to mixed inoculum has been reported previously [33, 34], and it is thus often used in commercial products.

 A similarly strong correlation (*r* = 0.70) between arbuscular colonization (%) and onion yield (tons/ha) as in our case was found in an extensive survey of onion under conventional management conducted in the Netherlands [14], but the correlation was found to be much lower (*r* = 0.47) for onion grown in a field under organic management.

 AMF directly or indirectly affects the soil ecosystem in different ways through multiple interactions with other soil organisms. Although they are not saprotrophs, AMF can enhance the rate of decomposition of organic material [35], indirectly influencing decomposition through interactions with other soil microorganisms [36]. Various interactions between AMF and saprotrophic fungi have been reported. *Trichoderma* in particular is now in the centre of attention because of its antagonistic effects against root pathogens and a possible synergistic interaction with AMF that helps promote plant growth. This synergy in the interaction appears to be very genotype-specific, however. Tiunov and Scheu [37] showed in their study that *G. mosseae *BEG12 suppressed the abundance of *Trichoderma harzianum *Rifai and *Exophiala *sp. while promoting the development and abundance of *Ramichloridium schulzeri *(Sacc.) de Hoog. *Trichoderma* genotypes exhibiting synergistic effects with AMF on growth of mycorrhizal plants have already been identified. Camprubi et al. [11] reported synergistic effects between *G. intraradices* and *T. aureoviride* on the growth of *Citrus reshni* in organic substrates. Tchameni et al. [12] observed positive effects of a mixture of two different mycorrhizal fungi (*Gigaspora margarita* and *Acaulospora tuberculata*) with the PR11 strain of *Trichoderma asperellum* promoting the growth of cacao trees. This mixture also induced resistance of cacao tree against the soil-borne pathogen *Phytophthora megakarya*.

 The saprotrophic fungi used in the present study were selected because they are efficient saprotrophic decomposers of wood and litter. To our knowledge, this is the first time that such a combination of saprotrophic basidiomycete fungi, preinoculated on bark chips, has been used together with AMF inoculation to improve vegetable plants. It has been reported that *A. praecox* and other basidiomycetous decomposers *in vitro* under intermediate nitrogen supply promoted carbon mineralization and induced high levels of ligninase activity in *A. praecox* cultures grown on wood of black spruce (*Picea mariana* (Mill.) Britton, Sterns & Poggenb.) [38]. This property of saprotrophic basidiomycetes, which helps plants retrieve nutrients from organic matter supplied in the form of woody material, thus may be utilized under conditions of low-input production schemes.

 The source of organic matter that is used as fertilizer in crop production may affect the achieved mycorrhizal responsiveness remarkably. Organic matter amendment in the form of ground leaves was found to lower the dry weight of shoots of AM cucumber plants compared to non-AM plants [39]. In the form of dried stems, it led to an increase in biomass of AM plants compared to non-AM plants of the tropical crop *Desmodium ovalifolium *L. [40]. Wheat bran amendment resulted in lowered biomass of non-AM tomato plants, but the biomass increased remarkably in plants treated either with a single inoculum containing AMF and the saprotrophic fungus *Clonostachys rosea* or a dual inoculation, which increased plant growth synergistically [41].

 Plant genotype selection for reduced functioning of AM symbiosis under agricultural intensification was postulated a long time ago [42]. A recent genetic analysis of *Allium* species suggests that modern onion breeding does not select against the response to AMF [31], as has been suggested before for other cultivated species, for example, wheat [43]. In the present study, we therefore did not focus on genotypic specificity of host-plant microbe combinations. We instead focused on *A. cepa* cv. “ALICE”, a genotype that is commonly used in high-input systems in the Czech Republic. Our results suggest promising applicability of the tested cultivation technology in commercial field production and small-scale cultivation alike.

 During the last decades, improvements of nutritional quality brought about by microbial treatments, mycorrhiza in particular, have received increasing attention. Nell et al. [34], for example, reported positive effects of the same mixed inoculum as the one used in this study (M1) on the production of secondary metabolites (sesquiterpenic acids) by *Valeriana *sp. Recent studies also show that AM plants can contain higher amounts of nutritionally important elements. AMF inoculation, for example, significantly increased the total dry weight, leaf P, K, Ca, Mg, Fe, Cu, and Mn contents in trifoliate orange (*Poncirus trifoliata *L. Rafin.) seedlings [44], or Cu and Fe contents in lettuce [45]. Increases in K, Ca, Mg, and Fe content were also found to be an effect of dual inoculation with AMF and saprobic fungi [46]. Researchers have recently recorded positive effects of AM on the content of different groups of compounds with antioxidative properties in harvested parts of crop plants, for example, organosulfur compounds in bunching onion [47], anthocyanins, carotenoids, and, to a lesser extent, phenolics in lettuce [45]. Arbuscular mycorrhizal fungi may support the production of organosulfur compounds under field conditions. In our study, we measured only the total antioxidant capacity, not specific compounds or chemical species, so we could not determine which species the observed increase should be ascribed to.

 AMF inoculation might become very important for sustainable agronomical management, especially in cases when the efficiency of native inocula is poor [48]. Positive effects of AM inoculation with nonnative species on the growth and mycorrhizal colonization of onion plants were observed in five previous field experiments [49]. In last decades, the mycorrhizal industry has been developing an entire range of mycorrhizal products although the concern remains about inoculum quality [50]. An ongoing debate remains whether introduced AMF can survive in a form in which mycorrhizal symbionts are efficient in natural field conditions. Sequencing of fungal ITS has already provided several lines of evidence about the persistence of inoculated AMF after 2 years suggesting [20]. Another concern in application of AMF inoculants is about lowering AMF diversity in target ecosystems caused by outcompeting indigenous fungi by introduced nonnative fungal strains. Applications of tuned AM inocula derived from isolated native AMF strains from a particular ecosystem could overcome this problem and have been already the strategy of some commercial companies [5, 6].

## 5. Conclusions

 Our results support the conclusion that there is a synergistic effect between dual microbial inoculation containing both AMF and saprotrophic fungi supplied together with organic matter. This ecological way of cultivation can lead to improvement of the parameters of onion plants as well as their nutritional value in sustainable production. Proper tuning of responsive genotypes of host plants, AMF, and saprotrophic fungi can bring not only a biomass increase but can also lead to improvement of nutritional quality. We believe that such synergistic dual fungal inoculations involving mycorrhizal and saprotrophic fungi together with organic matter supply have high potential for sustainable, environment-friendly production systems not only of onion but of crops in general.

## Abbreviations

AMF: Arbuscular mycorrhizal fungi
AM: Arbuscular mycorrhiza
TAC: Total antioxidant capacity.
